# Supplementary material for: Tubulin Polyglutamylation by TTLL1 and TTLL7 Regulate Glutamate Concentration in the Mice Brain
Source: Biomolecules. 2023 May 1;13(5):784. doi: 10.3390/biom13050784 (PMC10216738; doi:10.3390/biom13050784)
Supplement: Supplementary file 1 [file biomolecules-13-00784-s001.zip › biomolecules-2339756-supplementary.pdf]

biomolecules

MDPI

Article

Tubulin Polyglutamylation by TTLL1 and TTLL7 Regulate Glutamate Concentration in the Mice Brain

Yashuang Ping[1†], Kenji Ohata[1,2†], Kenji Kikushima[1,3], Takumi Sakamoto[1], Ariful Islam[1], Lili Xu[1], Hengsen Zhang[1], Bin Chen[1], Jing Yan[1], Fumihiro Eto[1], Chiho Nakane[1], Keizo Takao[4,5], Tsuyoshi Miyakawa[5,6], Katsuya Kabashima[1], Miho Watanabe[7], Tomoaki Kahyo[1,3], Ikuko Yao[1,3,8], Atsuo Fukuda[7], Koji Ikegami[1,9], Yoshiyuki Konishi[1,10], Mitsutoshi Setou[1,3,11,*]

1 Department of Cellular and Molecular Anatomy, Hamamatsu University School of Medicine, 1-20-1 Handayama, Higashi-ku, Hamamatsu, Shizuoka 431-3192, Japan
2 Department of Gastroenterology, Graduate School of Medicine, The University of Tokyo, 7-3-1 Hongo, Bunkyo-ku, Tokyo 113-0033, Japan
3 International Mass Imaging Center, Hamamatsu University School of Medicine, Hamamatsu, 1-20-1 Handayama, Higashi-ku, Hamamatsu, Shizuoka 431-3192, Japan
4 Department of Behavioral Physiology, Faculty of Medicine, University of Toyama, 2630 Sugitani, Toyama-shi, Toyama 930-0194, Japan
5 Genetic Engineering and Functional Genomics Unit, Frontier Technology Center, Graduate School of Medicine, Kyoto University, Kyoto 606-8501, Japan
6 Institute for Comprehensive Medical Science Division of Systems Medicine, Fujita Health University, Aichi 470-1192, Japan
7 Department of Neurophysiology, Hamamatsu University School of Medicine, Hamamatsu, 1-20-1 Handayama, Higashi-ku, Hamamatsu, Shizuoka 431-3192, Japan
8 Department of Biomedical Sciences, School of Biological and Environmental Sciences, Kwansei Gakuin University, 1 Gakuen Uegahara, Sanda, Hyogo 669-1330, Japan
9 Department of Anatomy and Developmental Biology, Graduate School of Biomedical and Health Sciences, Hiroshima University, 1-2-3 Kasumi, Hiroshima 734-8553, Japan
10 Department of Applied Chemistry and Biotechnology, University of Fukui, 3-9-1 Bunkyo, Fukui-shi, Fukui 910-8507, Japan
11 Department of Systems Molecular Anatomy, Institute for Medical Photonics Research, Preeminent Medical Photonics Education & Research Center, 1-20-1 Handayama, Higashi-ku, Hamamatsu, Shizuoka 431-3192, Japan
* Correspondence: setou@hama-med.ac.jp; Tel.: +81-053-435-2086; Fax: +81-053-435-2468
† These authors contributed equally to this work.

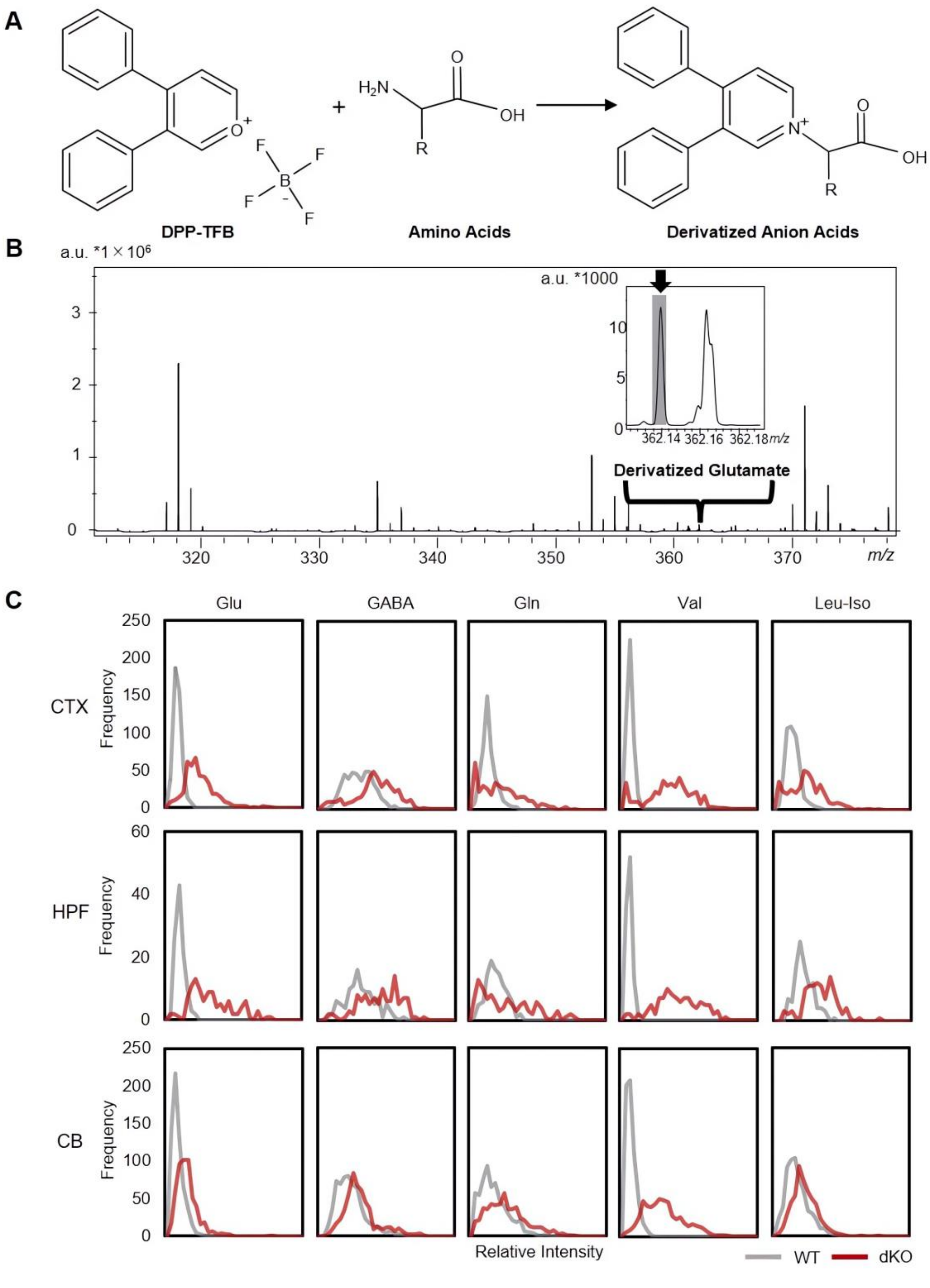

Figure S1. The derivatization of free amino acids by DPP-TFB and the relative intensity line graph of amino acid in the sagittal brain of mice sagittal brain. (**A**) Scheme of the derivatization of free amino acids by DPP-TFB. (**B**) The signal intensity of derivatized glutamate in brain tissues. (**C**) The frequency of the relative intensity of each spot in the selected regions of *Ttll1*/*Ttll7* double knockout and WT mice brains are represented in line graphs. Abbreviations: Glu: glutamate; GABA: γ-aminobutyric acid; Gln: glutamine; Val: valine; Leu-Iso: leucine-isoleucine; dKO: *Ttll1*/*Ttll7* double knockout.

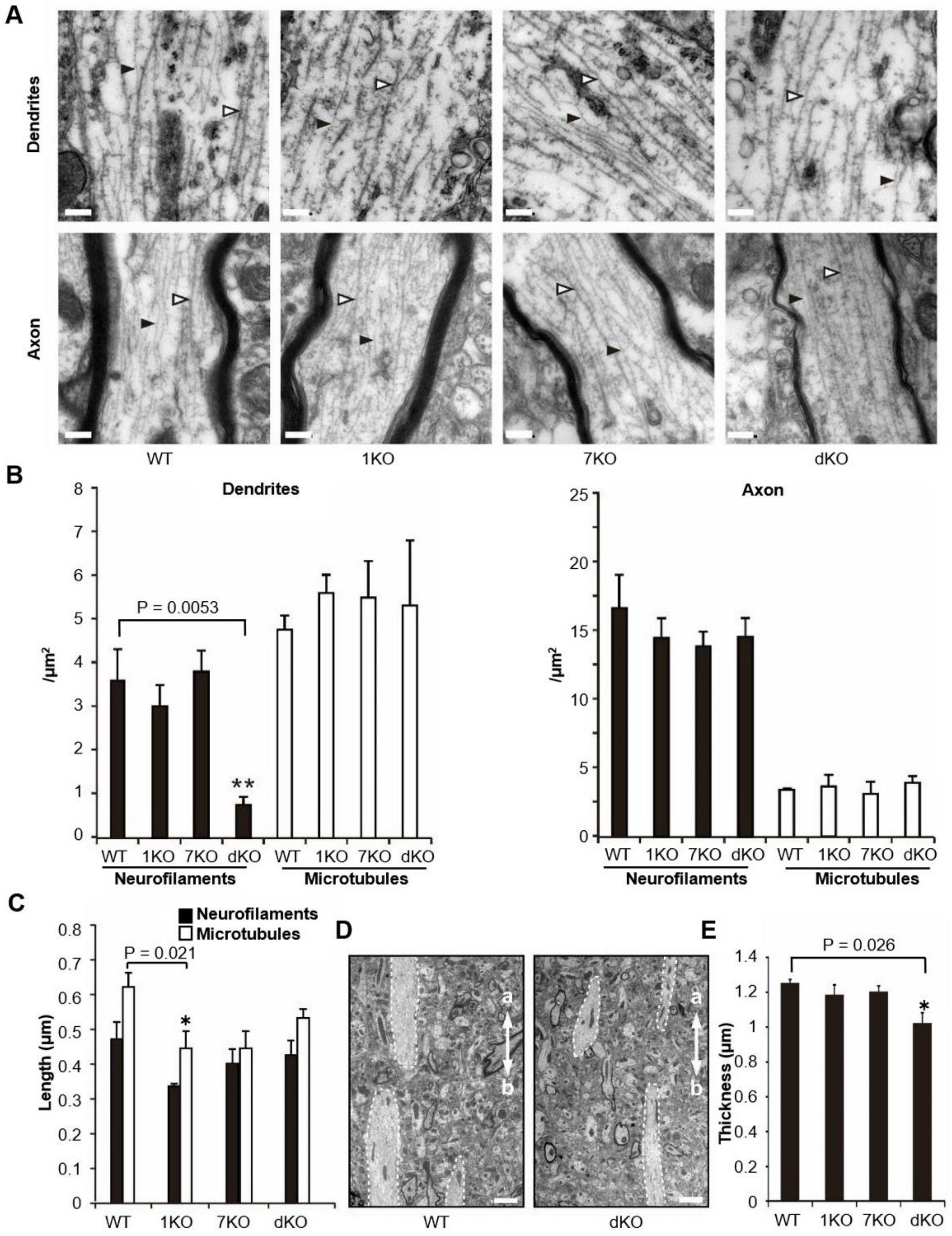

Figure S2. Electron microscopic observation of neurofilaments and microtubules in axons and dendrites of *Ttll* knockout mice. (**A**) Electron microscopic images of dendrites and axons from each transgenic mouse. Microtubules are pointed by white arrowheads, and neurofilaments are indicated by black arrowheads. Scale bars: 0.2 μm. (**B**) Densities of neurofilaments and microtubules in dendrites or axons were counted from the microscopic images of the cerebral cortex at layer II/III from each transgenic mouse. $p < 0.01$, denoted by **, (WT: 7 axons, 9 dendrites, n = 4 mice; 1KO: 7 axons, 6 dendrites, n = 3 mice; 7KO: 6 axons, 10 dendrites, n = 3 mice; dKO: 8 axons, 15 dendrites, n = 4 mice). (**C**) Neurofilament and microtubule lengths were calculated from EM images. $p < 0.05$, denoted by *. (**D**) Images of the dendritic shafts from wild type and *Ttll1*/*Ttll7* double knockout mice. Dendrites regions are indicated by dashed lines and measured at the widest region. a: apical direction; b: basal direction. Scale bar 2 μm. (**E**) The thickness of dendritic shafts from *Ttll1*/*Ttll7* double knockout mice was significantly reduced with $p < 0.05$, denoted by * (WT: 111 dendritic shafts, n = 3

mice; 1KO:127 dendritic shafts, n = 3 mice; 7KO: 116 dendritic shafts, n = 3 mice; dKO: 132 dendritic shafts, n = 3 mice). Data represented as mean ± SD in B, C, F, ANOVA followed by Dunnett's test. Abbreviations: 1KO: *Ttll1* knockout; 7KO: *Ttll7* knockout; dKO: *Ttll1*/*Ttll7* double knockout.

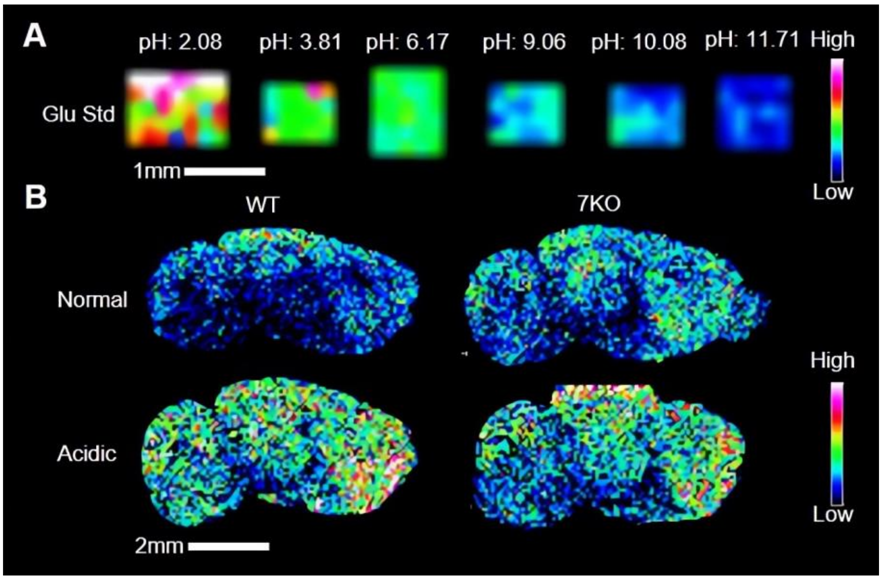

Figure S3. The detection efficiency of MALDI IMS for glutamate was influenced by pH. (**A**) Glutamate standard at different pH were detected by MALDI IMS, and the signal intensity of glutamate standards decreased with increasing pH. (**B**) Under normal conditions, glutamate concentrations in the brain of *Ttll7* knockout mice were higher than WT, but similar under acidic conditions. Abbreviations: Glu Std: Glutamate standard; 7KO: *Ttll7* knockout.

Table S1. MRM transitions and MS parameters for the analytes and IS of 4000 QTRAP MS/MS system.

Analyte	Precursor ion (*m*/*z*)	Product ion (*m*/*z*)	DP (V)	EP (V)	CE (V)	CXP (V)
Glu	148.1	84.0	26	10	21	12
IS-Glu	154.2	89.1	46	10	25	12
DPP-Glu	362.1	231.8	106	10	39	16
DPP-IS-Glu	368.1	232.9	46	10	35	8

MRM transitions and MS parameters for the analytes and IS of 4000 QTRAP MS/MS system. Abbreviations: Glu: glutamate, IS-Glu internal standard glutamate, DPP-Glu: DPP-TFB derivatization glutamate, DPP-IS-Glu: DPP-TFB derivatization internal standard glutamate, DP: declustering potential, EP: entrance potential, CE: collision energy, CXP: collision cell exit potential.
